# Overcoming the Leakage and Contact Resistance Challenges in Highly Scaled PMOS and NMOS Carbon Nanotube Transistors

**DOI:** 10.1021/acs.nanolett.5c00005

**Published:** 2025-03-03

**Authors:** Hsin-Yuan Chiu, Nathaniel Safron, Matthias Passlack, Tzu-Ang Chao, Sheng-Kai Su, Po-Sen Mao, Chen-Han Chou, Han-Yi Huang, Guan-Zen Wu, Chien-Wei Chen, Chi-Chung Kei, Wen-Hao Chang, H.-S. Philip Wong, Iuliana P. Radu, Gregory Pitner, Chao-Hsin Chien

**Affiliations:** † Institute of Electronics, National Yang Ming Chiao Tung University, Hsinchu 30010, Taiwan; ‡ Corporate Research, Taiwan Semiconductor Manufacturing Company, Hsinchu 30075, Taiwan; § Corporate Research, Taiwan Semiconductor Manufacturing Company, 2851 Junction Avenue, San Jose, California 95134, United States; ∥ Department of Electrophysics, National Yang Ming Chiao Tung University, Hsinchu 30010, Taiwan; ⊥ Taiwan Instrument Research Institute, National Applied Research Laboratories, Hsinchu 30076, Taiwan

**Keywords:** low-dimensional materials, carbon nanotube, transistor, high performance, low leakage, on/off ratio, doping, contact resistance

## Abstract

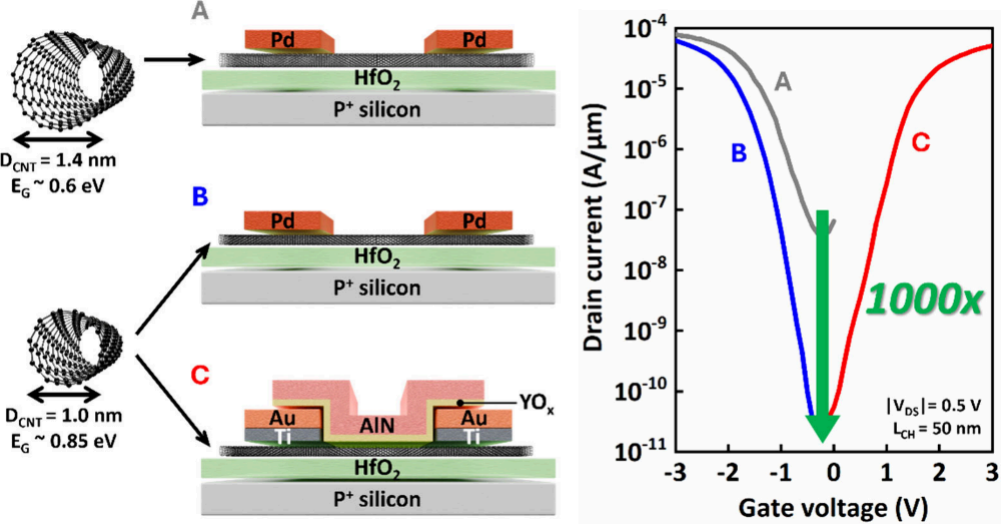

In this work, we address the off-state leakage current challenge, while simultaneously demonstrating high drive current per CNT, in NMOS and PMOS carbon nanotube field-effect transistors (CNFETs). Increasing the bandgap from 0.6 to 0.85 eV reduces the minimum current from 10^–8^ A/μm to 10^–11^ A/μm at *V*_DS_ = −0.5 V with a channel length of 50 nm. By utilizing titanium as the contact metal and the YO_*x*_/AlN solid-state electrostatic doping technique, isoperformance NMOS and PMOS are demonstrated in large bandgap CNFETs. We examined the contact properties of large bandgap CNT FETs by measuring the contact barrier height and report the contact resistance with contact lengths scaled down to 18 nm. Projections for high-density CNT arrays indicate promising potential for using large bandgap CNTs as channel materials in high-performance and low-power applications.

Intolerable off-state leakage current is a challenge in carbon nanotube field-effect transistors (CNFETs), in particular at drain-source voltages (*V*_DS_) exceeding 0.5 V.^1−8^ This is because most state-of-the-art CNFETs utilize Carbon Nanotubes (CNTs) with an energy bandgap (*E*_G_) equal to or smaller than 0.6 eV, in which exaggerated excellent on-state current above 1 mA/μm at *V*_DS_ = −0.5 V is usually claimed without addressing this off-state issue.^3,4,6−10^ The CNFET scalability is also promising with multiple demonstrations of channel length below 10 nm^11,12^ and the contact length down to 10 nm,^13^ validating the potential of CNT to replace silicon as the channel material for high-performance, highly scaled transistors in the front-end of line.^14,15^ However, the drawback of utilizing small bandgap CNTs is clear: Most high-performance CNFETs display an unacceptably high leakage current minimum above 100 nA/μm operating at −0.5 to −0.7 V *V*_DS_,^3,4,6−8^ whereas Silicon transistors can operate with a leakage current below 10 nA/μm.^16^ The off-state leakage problem due to band-to-band tunneling^1,2^ in MOSFETs (or Schottky tunneling in Schottky barrier FETs) certainly will result in uncompetitive static power and energy efficiency. Techniques to suppress the leakage current include adding an extra gate at the drain side to increase the tunneling barrier for carriers in the transistor off-state^17,18^ and introducing an asymmetric gate structure with an air gap between the gate and drain.^19,20^ However, the extra gate method has significant area and cost penalties and the asymmetric structures are not VLSI compatible in advanced technology nodes since they cannot be made with the typical self-aligned fabrication below resolution limits of lithographic patterning.^21^ Recent research on single-CNT transistors indicates that increasing CNT bandgap (*E*_G_ ∼ 0.85 eV) is another effective approach to suppress leakage without complicating the device architecture.^1,2^ Simulation results indicate that large-bandgap CNTs exhibit better subthreshold swing and lower off-state current (*I*_off_) in ultrascaled CNT MOSFETs with a gate length of approximately 12 nm compared to small-bandgap CNTs due to suppressed source-to-drain tunneling.^14,22^ However, increasing CNT bandgap raises questions on its impact on the CNT performance trade-offs, and whether this strategy is effective in both PMOS and NMOS.

In this paper, we highlight the potential of large bandgap ∼0.85 eV carbon nanotubes by demonstrating (i) 3 orders of magnitude reduced leakage current, compared to those with ∼0.6 eV bandgaps. (ii) Symmetric high performance in p-type and n-type CNFETs by integrating solid-state dielectric doping and contact engineering with large bandgap CNTs, attaining 4 μA/CNT and >6 orders of on/off ratio at |*V*_DS_| = 0.5 V and 50 nm of channel length (L_CH_). (iii) Finally, we report quantitative contact resistance for single-CNT N/PFETs with an 0.85 eV bandgap, including barrier height measurements and evaluations of contact resistance at 18 nm contact lengths. These results confirm that large bandgap CNTs are optimal for high-performance and low-power applications and should be utilized in future CNT electronics development.

For single-walled semiconducting CNTs, the bandgap is inversely related to the CNT diameter (*d*_CNT_), as shown in Figure 1a. In this study, we focus on transistors fabricated using CNT populations with bandgap distributions centered around 0.6 and 0.85 eV. The high semiconducting purity CNTs were prepared via polymer sorting methods from commercially available CNT powders: arc-discharge CNTs (Carbon Solutions, Inc., AP-CNT, diameter 1.2–1.6 nm) and HiPco CNTs (ATOM, HiPco, diameter 0.8–1.2 nm). As depicted in Figure 1b, the absorption spectrum of the HiPco CNT shows clear S11 and S22 peaks, while metallic M11 peaks are absent. Gaussian fitting of the S11 peaks, as illustrated in the inset of Figure 1b, was used to determine the chirality distribution and verify the bandgap range quantitatively.^23^ The bandgap calculations were based on the relationship using a tight-binding approximation: *E*_G_ ≈ 0.85/*d*_CNT_.^24^ By calculating the area ratio of each fitted Gaussian peak, the cumulative distribution plot (CDF) of the bandgap is shown in Figure 1c, where the median bandgap is determined to be 0.85 eV for HiPco CNTs. Additional characterization of the arc-discharge CNTs is provided in Supplementary Section 1. With the sorted CNT solution, the CNTs were deposited onto a substrate composed of 10 nm of HfO_2_ on heavily p-doped Si using an immersion method. The density of the deposited HiPco CNTs was measured to be approximately 13 CNTs per micrometer using Atomic Force Microscopy (AFM), as shown in Figure 1d.

**Figure 1:**
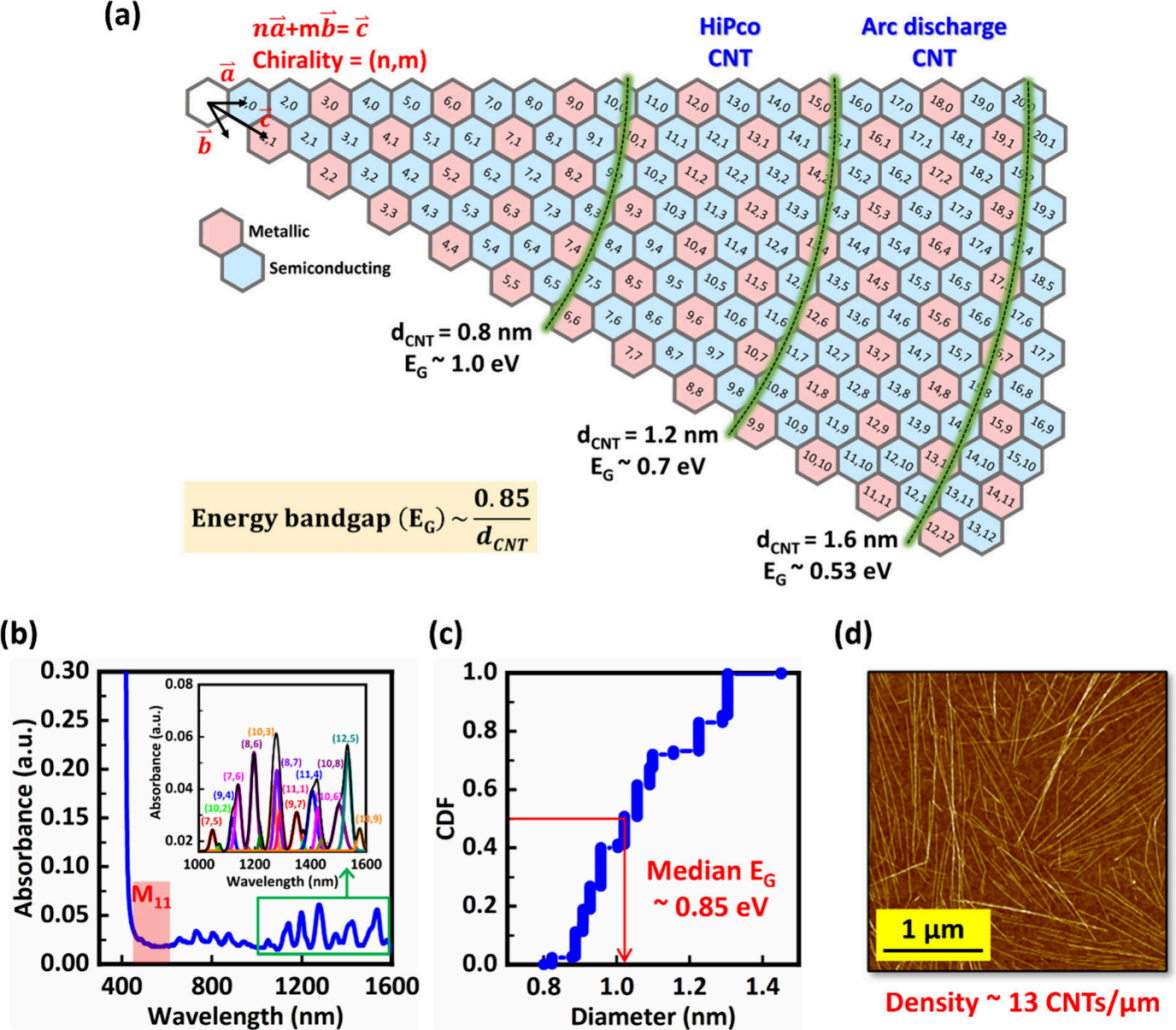
(a) Chirality table for single-walled carbon nanotubes. The primary diameter ranges for HiPco CNTs and Arc discharge CNTs are indicated in the plot. (b) Absorption spectra of large bandgap HiPco CNTs utilized in this work. Inset: Fitting of the absorption spectra of the S11 peak of HiPco CNTs. (c) Cumulative distribution function of the diameter of HiPco CNTs with a median value of ∼1.0 nm. The corresponding energy bandgap = 0.85 eV was calculated using tight binding approximation. (d) AFM image of HiPco CNTs deposited on the HfO_2_ substrate with density ∼13 CNTs/μm.

Back-gate CNFETs were fabricated using CNTs enriched with two distinct bandgap sizes, with median bandgap ≈0.6 and 0.85 eV respectively. The devices have a 50 nm channel length verified by scanning electron microscopy (Figure S2) and utilize palladium (Pd) as the source and drain contact metal, as shown in Figure 2a. The off-state and on-state band diagram of the p-type CNT back-gate Schottky-barrier field-effect transistor (SBFET) was analyzed through TCAD modeling, as shown in Figure 2b and 2c respectively. In SBFETs, switching occurs via modulation of the barrier at the contact-channel junction. The minimum leakage current is primarily governed by ambipolar tunneling at the drain contact. Therefore, a larger bandgap indicates that more energy is required for electrons to undergo thermionic emission over the Schottky barrier. Additionally, for the same gate-induced charge density in the CNT a larger bandgap accompanies larger Schottky barrier width, which decreases the ambipolar tunneling probability through the barrier. These factors help to reduce the electron injection when the CNFET is in the off state.

**Figure 2:**
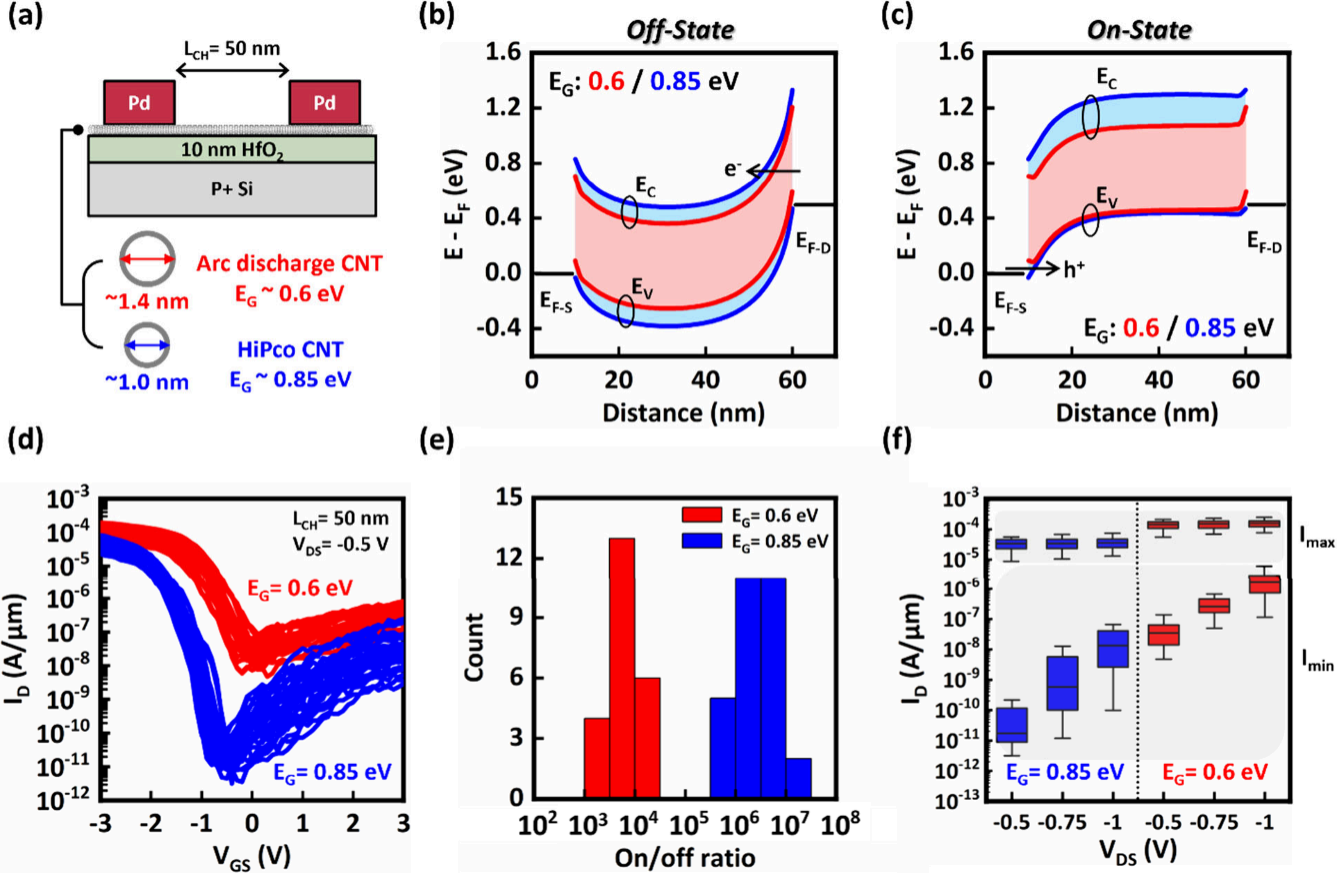
(a) Illustrated device structure for *I*_min_ comparison between large and small bandgap CNT channels. The channel length = 50 nm. Band diagram illustration for (b) off-state and (c) on-state of p-type CNFETs with 0.6 and 0.85 eV bandgaps. Electrical parameter comparisons between p-type CNFETs enriched with two different bandgaps of CNT channels for (d) transfer characteristics, (e) the on/off ratio, (f) the summary of *I*_max_ versus *I*_min_ measuring under *V*_DS_ = −0.5 V to −1 V.

Figure 2d and 2e show a significantly enhanced on/off ratio in the devices with larger bandgap CNTs, measured at *V*_DS_ of −0.5 V. Although hysteresis is present in the devices as shown in Figure S3, it does not impact the conclusion regarding *I*_min_, as it is independent of the gate sweeping direction. The difference in *I*_min_ observed at different *V*_GS_ can be attributed to bandgap differences, where the switching behavior of a Schottky barrier FET is governed by the potential barrier at the contact.^2^Figure 2f illustrates the relationship between maximum current (*I*_max_) and minimum current (*I*_min_) for CNFETs with varying bandgap sizes. At *V*_DS_ = −0.5 V, the data shows that *I*_min_ improves exponentially by more than 1000 times when the bandgap increases from 0.6 to 0.85 eV, while *I*_max_ decreases linearly by approximately five times due to the impact of the larger bandgap on the hole transport as described in Figure 2c. It is important to note that the reduction in *I*_max_ can also be partially attributed to the difference in the CNT density. Arc discharge CNTs exhibit a density of 26 CNT/μm (Figure S1b), while HiPco CNTs have a lower density of 13 CNT/μm (Figure1d). After normalizing for the density difference, the actual decrease in *I*_max_ due to the increased bandgap is approximately 2.5-fold per individual CNT. The advantages of the larger bandgap become even more pronounced at higher *V*_DS_ due to the reduced tunneling barrier. Figure 2f further explores this trend by summarizing current levels at various *V*_DS_, increasing from −0.5 V to −1 V. While the minimum current increases with higher *V*_DS_ for both small and large bandgap CNFETs due to the increased Schottky tunneling,^2^ it is noteworthy that the minimum current at −1 V in large bandgap CNTs remains lower than that at −0.5 V in small bandgap CNTs. This underscores the benefits of employing CNTs with larger bandgaps. The dependence of subthreshold swing on *V*_DS_ for different bandgaps is further discussed in Supplementary Section 4.

We confirmed the significant benefits of large bandgap CNTs for low leakage PFETs, yet the development of corresponding NFETs using large bandgap CNTs has not been reported to date, presenting a crucial gap for CMOS applications. To address this, we combined contact metal work-function tuning and solid-state n-type doping techniques to fabricate large bandgap CNT NFETs with electrical performance comparable to their PFET counterparts. The structure of the back-gated CNT NFET is shown in Figure 3a and features titanium (Ti) as the source-drain contact metal, chosen for its lower work function of approximately 4.5 eV, which reduces the barrier height for electron transport from the contact to the channel. The Ti contact is passivated by 20 nm of gold (Au) to prevent oxidation from the top. The top layer dielectrics consisted of a yttrium oxide (YO_*x*_) spacer of 4 nm and a 40 nm aluminum nitride (AlN) dopant layer.

**Figure 3:**
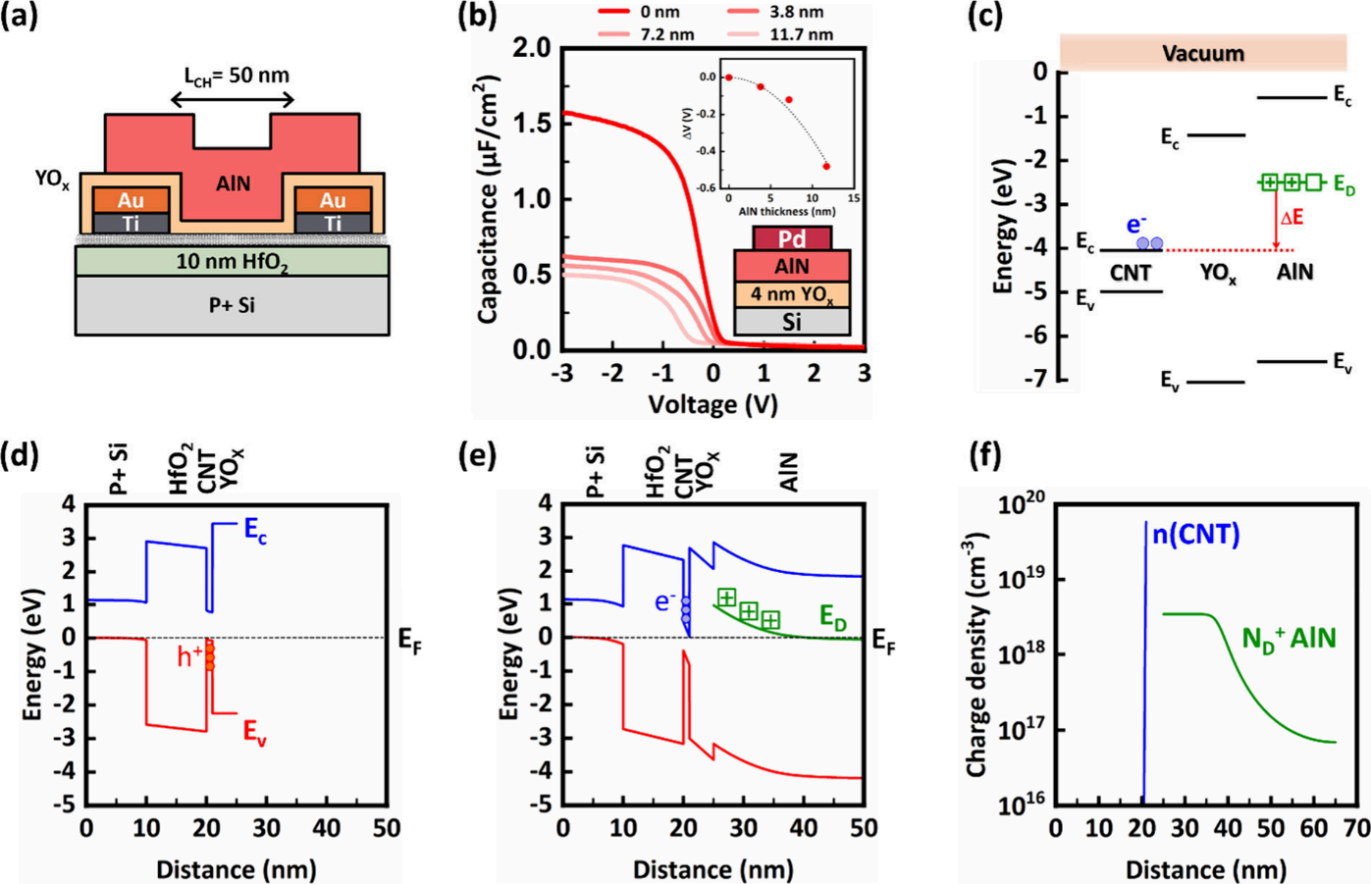
(a) Illustrative device structure for large bandgap CNT NFET, which utilizes titanium as source/drain contact metal and YO_*x*_/AlN dielectric doping. The channel length = 50 nm. (b) Capacitance–voltage characteristics for Si MOSCAP with various thickness of AlN. Inset: The measured voltage shift (Δ*V*) along the voltage axis for each condition using the curve with *t*_AlN_ = 0 as reference (symbols) and the illustration of the Si MOSCAP structure. (c) Illustration of energy band structures of large bandgap CNT, YO_*x*_, and AlN layers in separation. The AlN bulk donor level *E*_D_ is indicated. Energy band diagrams of the channel region (d) without AlN showing CNT p-type conductivity, and (e) with AlN showing conversion of the CNT conductivity to n-type due to donation of electrons from AlN donor states. (f) The volume density of positive charge on the AlN bulk level and the electron density of CNT as a function of distance.

In order to elucidate the doping mechanism and to quantify the AlN defect density, we fabricate Si MOSCAPs with various AlN thicknesses (*t*_AlN_ = 0, 3.8, 7.2, and 11.7 nm), as shown in Figure 3b. The AlN bulk donor density N_AlN_ was extracted by analyzing the shift along the voltage axis Δ*V* (Figure 3b, inset) with various AlN thicknesses to 3.4 × 10^18^ cm^–3^. Supplementary Section 5 provides a detailed discussion of N_AlN_ extraction. For the n-doping process to be effective, the donor state energy level within the bulk AlN must be higher than the conduction band-edge energy of the CNTs (Δ*E*) as indicated in Figure 3c. Self-consistent equilibrium solutions for the layers in contact are shown in the energy band diagrams in Figure 3 (d) without AlN layer, and (e) with AlN layer. In Figure 3d, charge equilibrium is provided by holes induced in the CNT compensating for the net negative ionized acceptor charge on the p^+^ Si surface. When the AlN is present (Figure 3e), the bulk AlN donor level moves above *E*_F_ and consequently becomes ionized near the CNT. The positive charge on the bulk AlN donor level forces electrons to accumulate in the CNT forming an electron channel. Therefore, the doping strength is expected to decrease with thinner AlN, as fewer donor levels remain in close proximity to the CNT to facilitate electrostatic doping.^25,26^ The positive charge volume density on the AlN bulk level and the CNT electron density are shown in Figure 3f as a function of distance. Here, the energy level of the ionized donor state is assumed to be situated 1.6 eV above the *E*_c_ of the CNT (Δ*E* = 1.6 eV). The YO_*x*_ serves three primary functions: (1) It acts as an adhesion layer for subsequent AlN deposition by ALD, as CNTs lack nucleation sites.^27,28^ (2) It prevents electron mobility degradation by acting as a barrier layer, reducing Coulomb scattering from charge impurities near the doping interface,^29^ which is also observed in materials like molybdenum disulfide (MoS_2_).^26^ (3) Further, the thickness of YO_*x*_ may be increased or decreased to tune doping strength.^29^ The entire NFET fabrication process complies with the stringent back-end-of-line (BEOL) CMOS process requirements, maintaining all procedures below the critical thermal budget of 400 °C.

Figures 4a and 4b display the symmetric behavior in the transfer, output, and transconductance curves. The ohmic contact behavior in both NFET and PFET configurations is evidenced by the linear characteristics of the output curves at a low drain voltage. The increase in *I*_D_ in the output curves when *V*_DS_ exceeds 0.7 V for nFETs is attributed to Schottky tunneling, and the corresponding band diagrams are presented in Supplementary Section 6. The stability of the devices is tested over 70 days with no degradation in the electrical performance, as detailed in Supplementary Section 7. Figure 4c summarizes the device DC metrics measured at |*V*_DS_| = 0.5 V. The on-current exceeds 4 uA/CNT with off-current below 2.3 pA/CNT for both NFET and PFET; this off-current level is 3 orders lower than the reported single-CNT transistors.^11^

**Figure 4:**
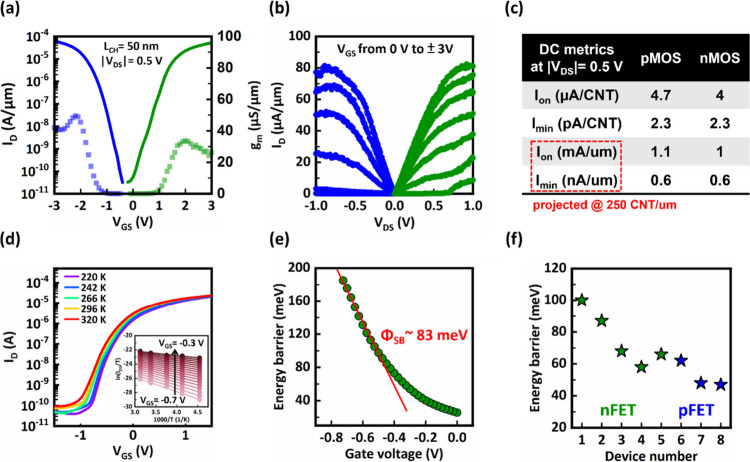
(a) N/P parity of large bandgap CNFETs in transfer characteristics (line) and transconductance (squares). The contact length (*L*_c_) is 200 nm. (b) Output characteristics of the large bandgap CNT NFET (green) and PFET (blue). (c) Summary table of the DC metrics at |*V*_DS_| = 0.5 V of the measured CNFETs. (d) Temperature dependence measurement of CNT NFET with *E*_G_ = 0.85 eV at *V*_DS_ of −0.05 V. Inset: The Arrhenius plot extracted from the corresponding temperature dependence measurement. (e) The electron energy barrier = 83 meV extracted from the slope of the Arrhenius plot. (f) Energy barrier distribution of CNT NFET (green) and PFET (blue) with *E*_G_ = 0.85 eV.

To study the contact properties, we extract the Schottky barrier height (SBH) using temperature-dependent *I*–*V* measurements and the thermionic emission equation^30^ at small *V*_DS_: *I*_D_ ∝ *T* exp(), where *T* represents the absolute temperature, *k* is the Boltzmann constant, and Φ_SBe_ denotes the electron Schottky barrier height. Figure 4d demonstrates the transfer characteristic of a large bandgap CNT NFET measured across a temperature range from 220 to 320 K. The drain current rises as temperature increases, indicating enhanced thermionic emission. For precise quantification of the SBH, the natural logarithm of *I*_D_/*T* versus 1/*T* (an Arrhenius plot) was constructed, as shown in Figure 4d, inset. The slope of a linear fit to this plot yields the effective SBH at various gate voltages, as shown in Figure 4e. The effective SBH is determined by analyzing the transition from a thermionic emission-dominated regime to one where tunneling mechanisms are prevalent. Figure 4f presents an evaluation across five NFETs, revealing a median SBH of 68 meV, slightly higher than those observed in PFETs with median SBH of 45 meV. Variability in the SBH across these devices may arise from differences in CNT chirality and bandgaps within the channel or from the metal work function differences for different metal surfaces facing the CNTs.

In the previous section, we demonstrated that the titanium (Ti) contact metal and AlN dopant layer on the channel yield symmetric electrical performance for CNT NFETs compared to PFETs, but the contact length (*L*_c_) in these transistors was 200 nm. The reduction of contact length remains crucial for advancing contact technology for increased transistor density. There are few reports on the contact resistance at highly scaled contact lengths for large bandgap CNT N/P FETs.^31^ Transitioning to the short-contact regime represents a critical threshold where contact resistance increases sharply, which is inversely proportional to *g*_c_ × *L*_c_.^32^ Therefore, we scaled the contact length to 18 nm in single-CNT field-effect transistors to investigate the relationship between contact resistance and contact length.^33^ The dimensions of the longest and shortest contacts were confirmed by high-resolution transmission electron microscopy cross sections, as shown in Figure S8. The total resistance can be simplified to the formula, and these components are defined in Figure 5a:
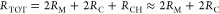
By measuring *R*_TOT_ and *R*_M_, the calculated value of *R*_C_ represents the upper bound of the actual contact resistance, assuming that the channel resistance is negligible in a CNFET with 50 nm L_CH_.^13,33,34^ To ensure that each transistor has a single carbon nanotube in the channel, the CNT solution was significantly diluted before being deposited on an HfO_2_/Pt substrate, achieving a density of approximately 1 CNT/μm. Furthermore, the active area was patterned with a channel width of 500 nm.

**Figure 5:**
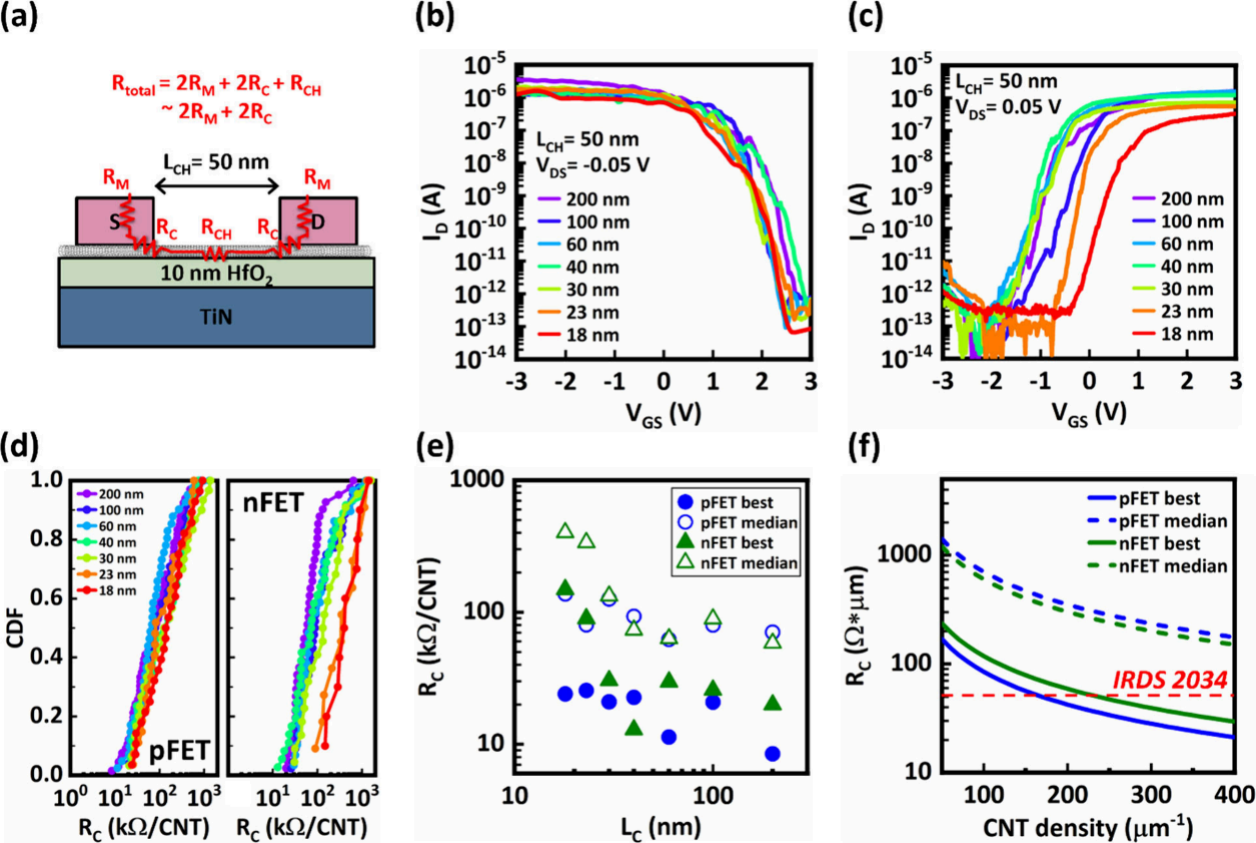
(a) Illustration of the overall resistance for a single CNT FET. (b) Transfer curves for the PFETs with the lowest resistance in each contact length at *V*_DS_ = −0.05 V. (c) Transfer curves for the NFETs with the lowest resistance in each contact length at *V*_DS_ = 0.05 V. (d) Cumulative distribution function of contact resistance for PFET (left) and NFET (right) with contact length scale down to 18 nm. (e) The best and median *R*_C_ values from this work. (f) *R*_C_ projection to dense array CNTs. The *R*_C_ value from IRDS 2034 is normalized to the total number of stacked silicon sheets.

Figure 5b and 5c show the *I*_D_–*V*_GS_ curves for the CNT PFETs and NFETs with the lowest resistance at each contact length. The contact resistance (*R*_C_) was determined from the drain current measurements at the maximum gate voltage, ensuring that 2*R*_C_ significantly exceeds the channel resistance. Figure 5d reports the distribution of *R*_C_ values for various contact lengths, encompassing both the shortest contacts and the lowest resistance of large-band gap CNFET contacts reported to date. The best and median *R*_C_ values are summarized in Figure 5e. SEM analysis confirms that devices with the best *R*_C_ contain a single nanotube within the channel (Figure S9). Given that the nanotube is likely not perfectly perpendicular to the contact electrode edge, the actual *L*_C_ for these top-performing devices was measured via SEM and is presented in Supplementary Section 9. For PFETs, the best contact resistance of 8.4 kΩ per CNT was observed at a 200 nm contact length, with a slight increase to 25 kΩ per CNT as the contact length scaled down to 23 nm. The trend of increasing *R*_C_ for contacts shorter than 30 nm aligns with the distributed contact model for reducing contact length discussed earlier. In contrast, NFETs showed the best contact resistance of 13 kΩ per CNT at longer contact lengths, which substantially increased to 149 kΩ per CNT when the titanium (Ti) contact length was reduced to 18 nm. This increase is due to the oxidation of Ti on both sides (Figure S10), effectively reducing the contact length. To address this issue, modifying the processing environment to an oxygen-free atmosphere or incorporating protective encapsulation layers could be effective solutions. Based on the best and median *R*_C_ values from this study, it appears feasible to meet the 2034 IRDS roadmap goal for contact resistance, as shown in Figure 5f. Here the contact resistance from IRDS 2034 was normalized to a single Silicon nanosheet. The on-current performance of dense array large *E*_G_ CNT FETs is assessed using the Monte Carlo method with an assumption of 250 CNTs/μm, as depicted in Supplementary Section 11. The maximum current surpasses 800 μA/μm at |*V*_DS_| = 0.5 V, except for the nNET with an 18 nm contact length. Furthermore, all devices exhibit an on/off ratio of 5 orders of magnitude. These findings suggest that large bandgap CNT is a viable candidate for high-performance, low-leakage, and low-power channel material.

In conclusion, our research demonstrated a 3 orders of magnitude improvement in the on/off ratio for CNFETs utilizing large bandgap CNTs at |*V*_DS_| = 0.5 V. This significantly surpasses the leakage performance of small bandgap CNTs reported in previous studies and uses methods which are compatible with VLSI fabrication in the BEOL. This improvement was achieved with a substantial reduction in minimum current and only a slight linear decrease in maximum current. We demonstrated n-type FETs with titanium as the contact metal and solid-state doping for large bandgap CNTs. The electrical performance of comparable NFETs and PFETs remained stable over a 70 day testing period, showing no signs of degradation. Temperature-dependent measurements confirmed minimal barrier formation at the contact region for both the NFETs and PFETs. Moreover, we reported contact resistances for large bandgap CNT NFETs and PFETs with contact lengths ranging from 18 to 200 nm. These results address a key concern regarding the contact resistance of highly scaled CNFETs with a larger CNT bandgap. Our statistical *R*_C_ data at each contact length and Monte Carlo simulation provide an accurate projection of achievable *R*_C_ and on current for dense array large bandgap CNTs in high-performance applications. For demonstrations of high-performance highly scaled CNFETs, future studies may utilize dense arrays of large bandgap CNT MOSFETs with the optimized contact metals and doping films from this work localized to spacer regions, such as Pd contact with WO_*x*_ dopant for PFETs and Ti contact with YO_*x*_/AlN dopant for NFETs. Additionally, exploring doping materials with low dielectric constants (<3.9) could further reduce gate-to-drain parasitic spacer capacitance, optimizing the intrinsic gate delay.

## Supporting Information Available

Material characterization of arc-discharge CNTs: absorption spectra and AFM data; SEM images for the long contact length (200 nm) devices; The hysteresis of the back gate CNFETs; The dependence of subthreshold swing on *V*_DS_ for different bandgaps; The detailed discussion of the AlN n-type doping mechanism; Off-state band diagrams of CNT Schottky barrier FETs; The stability of the CNT NFET; TEM images and EDX mapping for titanium contact with contact lengths of 200 and 18 nm; SEM images of devices with the best *R*_C_ for each contact length and corresponding contact length measurements and the Monte Carlo projections to dense array CNT FETs (PDF)
