# Supplementary material for: Overcoming the Leakage and Contact Resistance Challenges in Highly Scaled PMOS and NMOS Carbon Nanotube Transistors
Source: Nano Lett. 2025 Mar 3;25(10):3981–8. doi: 10.1021/acs.nanolett.5c00005 (PMC11907636; doi:10.1021/acs.nanolett.5c00005)
Supplement: Supplementary file 1 — nl5c00005_si_001.pdf [file nl5c00005_si_001.pdf]

Supporting information of

Overcoming the Leakage and Contact Resistance Challenges in Highly Scaled PMOS and NMOS Carbon Nanotube Transistors

Hsin-Yuan Chiu[1,2], Nathaniel Safron[3], Matthias Passlack[3], Tzu-Ang Chao[2,4], Sheng-Kai Su[2], Po-Sen Mao[2,4], Chen-Han Chou[1], Han-Yi Huang[1], Guan-Zen Wu[1], Chien-Wei Chen[5], Chi-Chung Kei[5], Wen-Hao Chang[4], H.-S. Philip Wong[2], Iuliana P. Radu[2], Gregory Pitner[3], Chao-Hsin Chien[1*]*

1 Institute of Electronics, National Yang Ming Chiao Tung University, Hsinchu 30010, Taiwan

2 Corporate Research, Taiwan Semiconductor Manufacturing Company, Hsinchu 30075, Taiwan

3 Corporate Research, Taiwan Semiconductor Manufacturing Company, 2851 Junction Avenue, San Jose, CA 95134, USA

4 Department of Electrophysics, National Yang Ming Chiao Tung University, Hsinchu 30010, Taiwan

5 Taiwan Instrument Research Institute, National Applied Research Laboratories, Hsinchu 30076, Taiwan

*gpitner@tsmc.com, *chchien@nycu.edu.tw

1. Material characterization of arc-discharge CNTs. The absorption spectrum of arc-discharge CNTs is shown in **Figure S1a**, with no metallic M11 peak detected. Unlike HiPco CNTs, the spectral fitting for arc-discharge CNTs is less precise due to the absence of cross-checking by PL maps[1]. As a result, the diameter distribution of the arc-discharge CNTs was characterized using atomic force microscopy (AFM), as shown in **Figure S1b**. The cumulative distribution function (CDF) of the diameters measured by AFM shows a median value of approximately 1.4 nm **(Figure S1c)**. The network CNT density difference between arc-discharge CNTs (26 CNT/um) and HiPco CNT (13 CNTs/um) could be attributed to the polymer (PCz) wrapping efficiency during the sorting process of high-purity semiconducting CNT solutions.

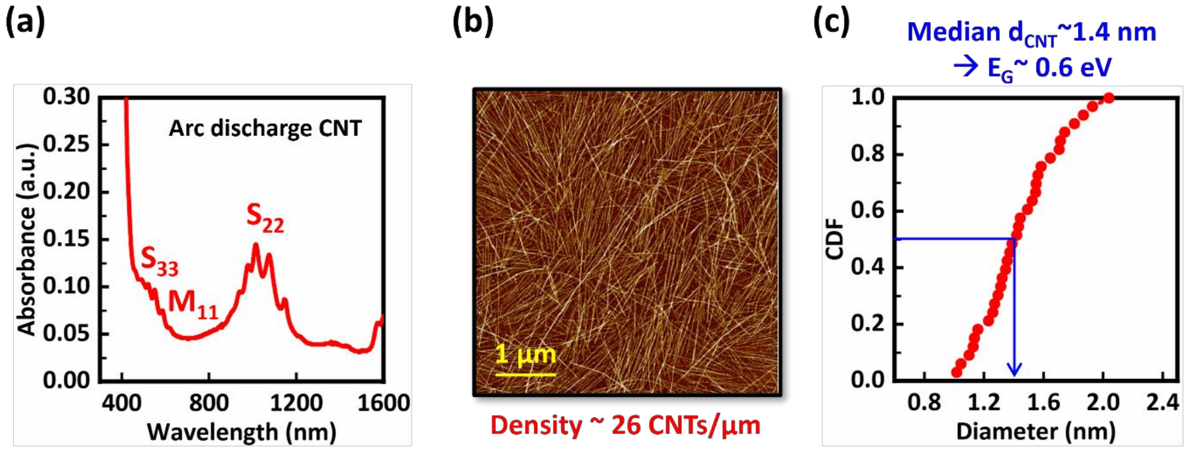

Figure S1. (a) Absorption spectra of arc-discharge CNTs utilized in this work. (b) AFM image of arc-discharge CNTs deposited on the HfO_2 substrate with density ~ 26 CNTs/µm. (c) Cumulative distribution function of the diameter of arc-discharge CNTs with a median value of

~1.4 nm. The corresponding energy bandgap = 0.6 eV was calculated using tight binding approximation.

2. SEM image of the CNFETs.

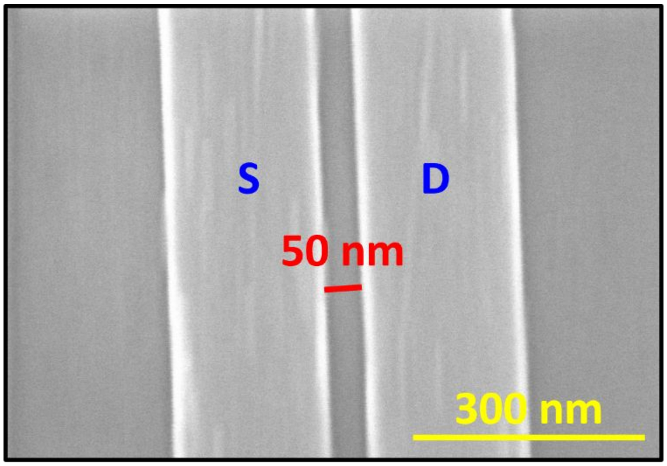

Figure S2. SEM image of the fabricated CNFETs. The channel length = 50 nm.

3. Hysteresis of the back gate CNFETs.

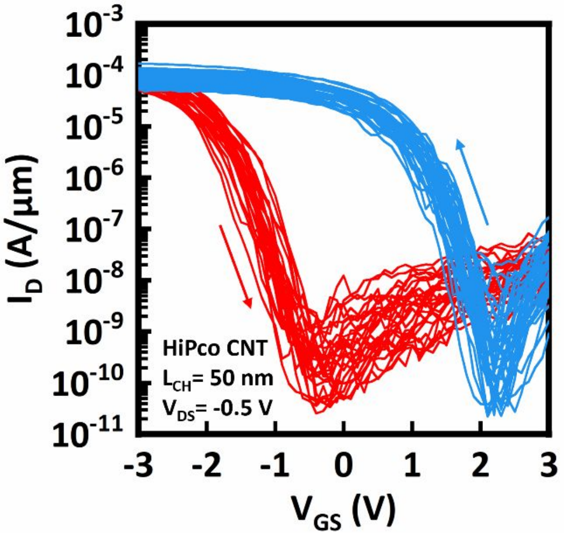

Figure S3. Transfer curves of back gate CNFETs with gate bias sweeping in both directions. The channel length = 50 nm.

4. The dependence of subthreshold swing on V_{DS} for different bandgaps.

The increase in SS at lower I_D for arc-discharge CNTs is attributed to ambipolar current. Under higher V_{DS}, the band bending at the drain side becomes more pronounced, leading to the onset of

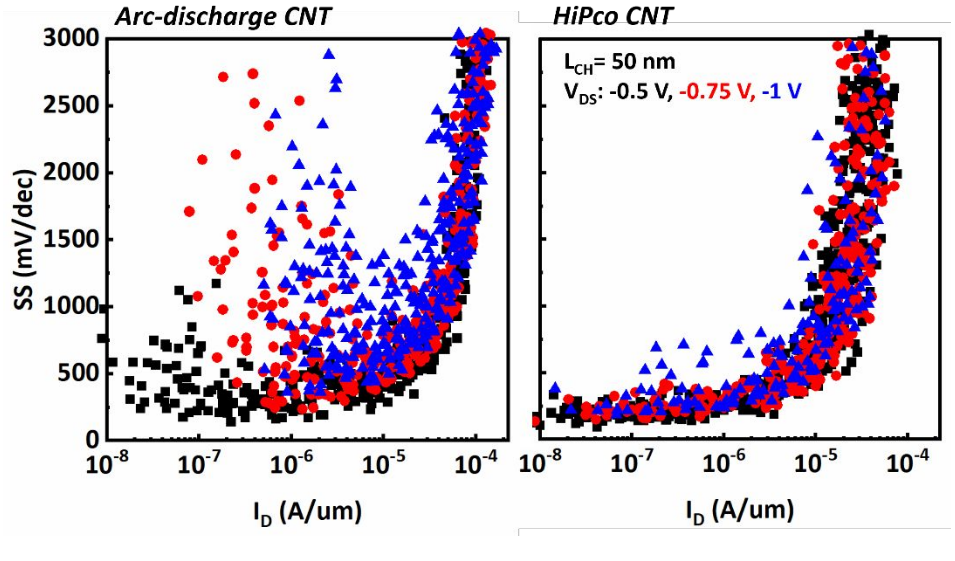

ambipolar leakage current. This effect is more significant in small-bandgap CNTs, as their shorter tunneling width enhances ambipolar tunneling. In contrast, we observe large-bandgap CNTs exhibit relatively less SS degradation under high V_{DS} due to their larger tunneling width, which suppresses ambipolar tunneling-induced leakage.

Figure S4. The change of SS as a function of V_{DS} for CNT with different bandgaps.

5. AlN N-type doping mechanism characterization.

The equation in **Figure S5** indicates that the flat band voltage (V_{FB}) is influenced by different charge components and their dependence on the equivalent oxide thickness (EOT):

1. Interface Charges ($Q_{interface}$): When interface charges dominate, V_{FB} exhibits a linear dependence on EOT. This is captured by the term EOT × [$-qQ_{interface}/(\kappa\varepsilon_0)$].

2. Bulk Charges (ρ_{high_k}): When bulk charges are dominant, V_{FB} shows a quadratic dependence on EOT, as indicated by the term $EOT^2 \times [-q\rho_{high\_k} / (2\kappa\varepsilon_0)]$.

3. Dipole Charges (Q_{dipole}): When dipole charges dominate, V_{FB} becomes independent of EOT, as seen in the term $-qQ_{dipole}/\varepsilon_0 \times [(d_2/\varepsilon_2) - (d_1/\varepsilon_1)]$.

This systematic behavior allows differentiation of the charge type and its spatial location by analyzing the dependence of V_{FB} on EOT. The shift in flat band voltage ΔV shown in **Figure 3b** is obtained using the shift of the C-V curves in the depletion region to exclude effects due to capture/emission of accumulation or inversion carriers close to the band edges. The best fit is obtained with a parabolic dependence of ΔV on t_{AlN} which indicates bulk traps as the source of dopant charge. From the equation, we set $b = -qN_{AlN}/(2\kappa\varepsilon_0)$ with q, N_{AlN}, κ and ε_0 being the unit charge, the AlN bulk donor density, the AlN κ-value (7.7), and the permittivity of vacuum, respectively. Fitting to the measured data points gives $b = -4\times10^{-3}$ V/nm^2 and $N_{AlN} = 3.4 \times 10^{18}$ cm^{-3}. For **Figure 3d, 3e**, and **3f**, the numbers used in the model are as follows. CNT: $E_C = -4.075$ eV, $E_V = -4.925$ eV. AlN: $E_C = -0.6$ eV, $E_V = -6.63$eV. The E_D position of AlN was assumed as -2.5 eV in the analysis, this brings E_C (CNT) close to E_F. The E_D position can be used to tune the electron density in the CNT. In reality, the defect levels may be spread out over some energy range.

$$V_{FB} = \text{EOT}\left[-q\frac{Q_{interface}}{\kappa\epsilon_0}\right] + EOT^2\left[-q\frac{\rho_{high\_k}}{2\kappa\epsilon_0}\right] - \frac{qQ_{dipole}}{\epsilon_0}\left[\frac{d_2}{\epsilon_2} - \frac{d_1}{\epsilon_1}\right]$$

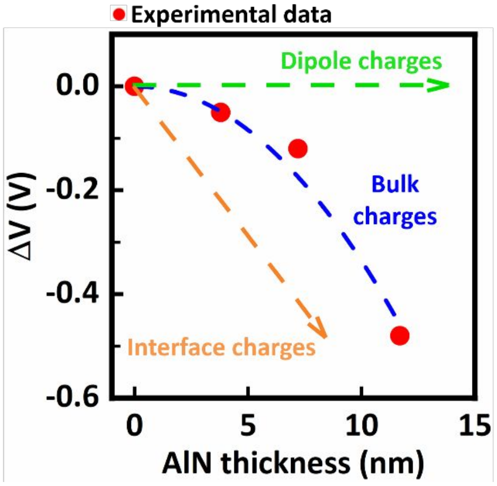

Figure S5. The correlation between flat band voltage and equivalent oxide thickness (EOT).

6. Band diagrams of Schottky barrier CNT nFETs and pFETs at various V_{DS} values in the off-state.

The increase in I_D (**figure4b)** at large V_{DS} for a fixed V_{GS} can be attributed to Schottky tunneling. **Figure S6** illustrates the band diagrams of Schottky barrier CNT nFETs and pFETs at various V_{DS} values in the off-state. As V_{DS} increases, the Schottky barrier near the drain becomes thinner, facilitating carrier injection into the nanotube and leading to an increase in leakage current.

In our study, the nFET (Ti contact, work function ~4.4 eV) has its Fermi level positioned closer to the midgap compared to the pFET (Pd contact, work function ~5.1 eV), as confirmed by the barrier height extraction shown in **Figure 4f**. Consequently, hole carriers in nFETs can tunnel from the drain at a lower V_{DS} than in pFETs.

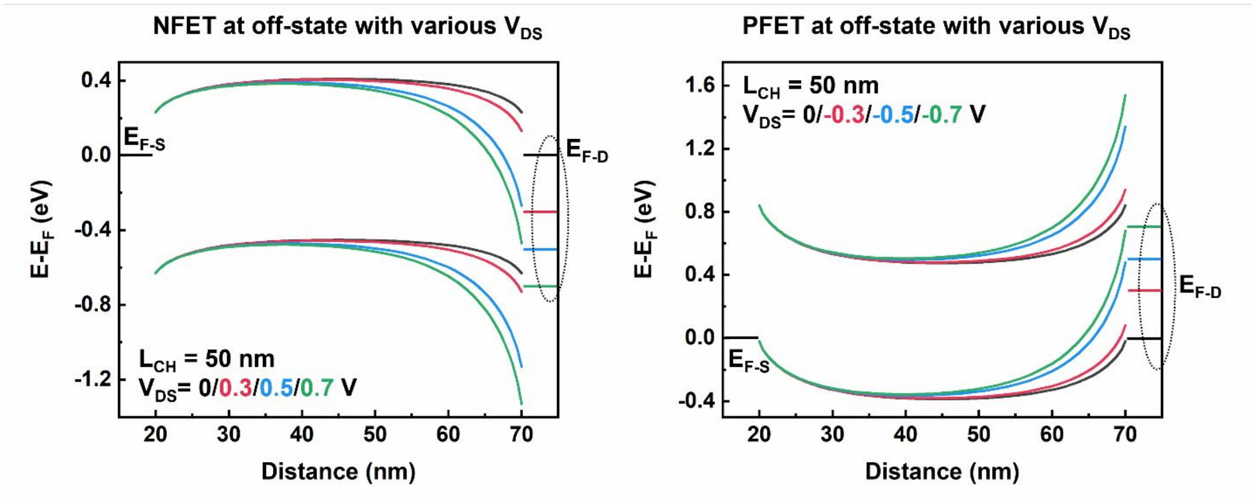

Figure S6. Band diagram illustration for nFET (left) and pFET (right) in the off-state with various V_{DS}.

7. The stability of the n-type doping for large bandgap CNT NFET.

The stability of the large-bandgap CNT NFET was evaluated 70 days post-fabrication on devices with L_{CH}=50 nm and 200 nm. The results reveal negligible variations in transfer characteristics, maintaining a 100% yield with virtually no change in maximum drain current ($I_{D,max}$) after 70 days across 50 devices for each channel length.

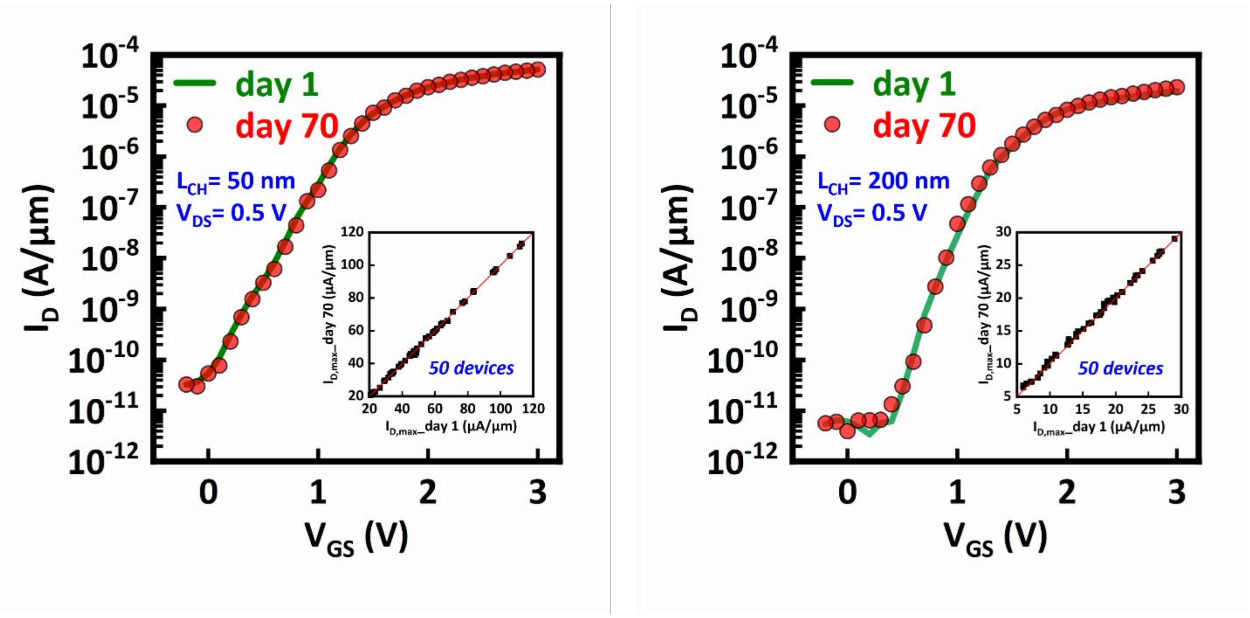

Figure S7. Transfer curves and statistical data of $I_{D,max}$ for the device as fabricated and 70 days post-fabrication.

8. High-resolution cross-section TEM images of the metal contact dimension for the longest and the shortest contacts presented in this study.

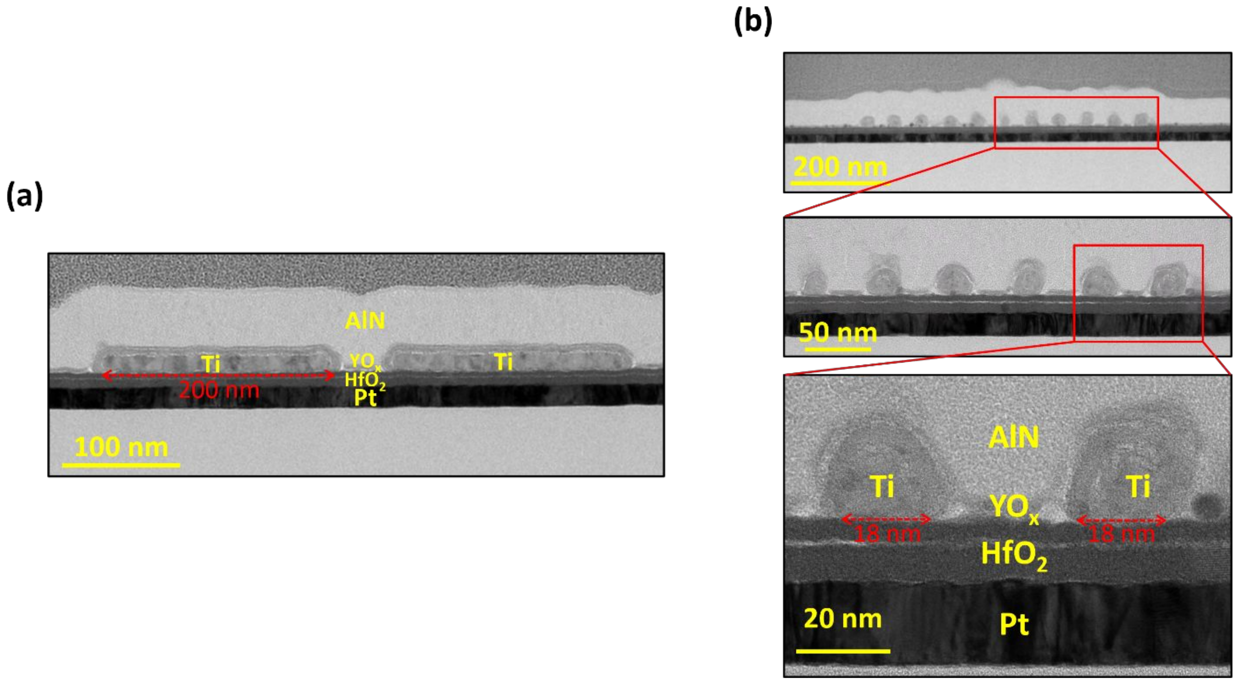

Figure S8. TEM images of the metal contact dimension for (a) the longest contacts =200 nm and (b) the shortest contacts = 18 nm.

9. SEM images of devices with the best R_C for each contact length and corresponding contact length measurements.

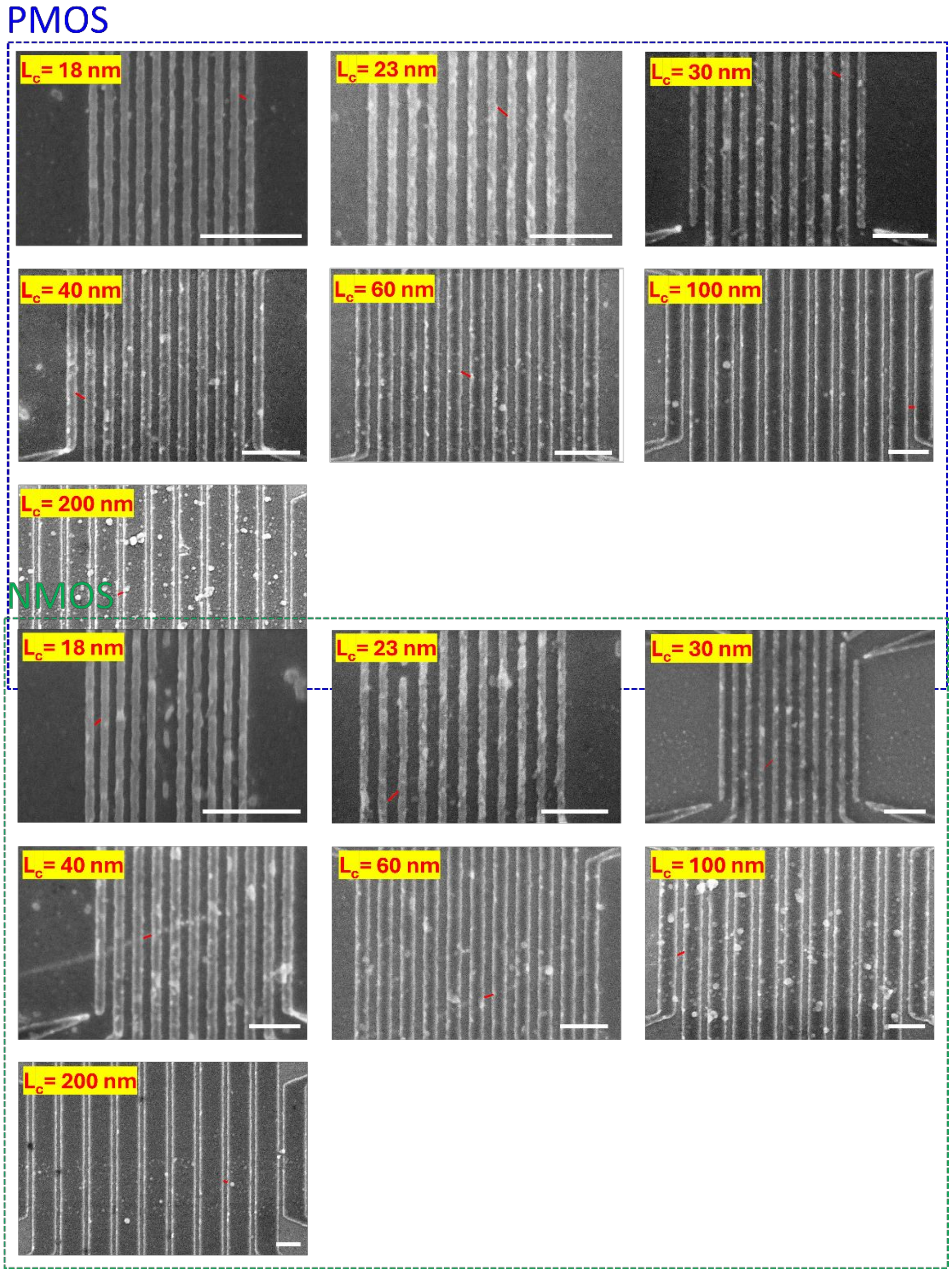

Ideally (nm)	Experimentally (pFET) (nm)	Experimentally (nFET) (nm)
18	18.8	24
23	25.6	34
30	31	35
40	42.5	41
60	61.3	65
100	100	106
200	208	209

Figure S9. SEM images of devices with the best R_C for each contact length and corresponding contact length. The CNT is highlighted in red and the scale bar = 200 nm.

10. EDX mapping for Ti source/drain metal with contact lengths of 200 nm and 18 nm.

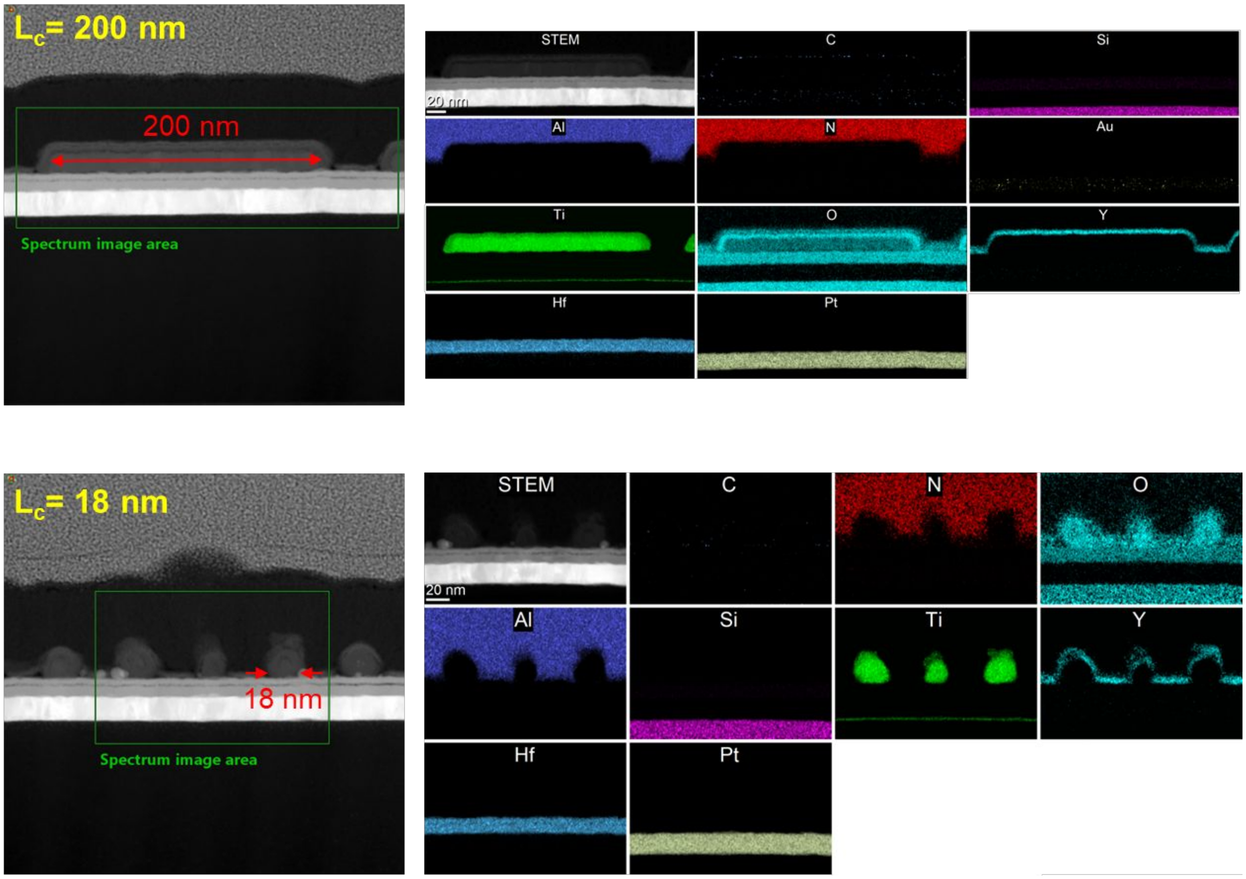

Figure S10. EDX mapping of the titanium contact. Top: At 200 nm contact length, a self-limiting TiO_x layer forms on the Ti contact surface. This dense oxidation layer prevents complete oxidation of the internal titanium. Bottom: For contact length = 18 nm, the titanium contact is fully oxidized to TiO_x due to the overlapping oxidation layers from both sides.

11. Dense array CNT projection by the Monte Carlo method. The single CNT I_D-V_{GS} data points are randomly selected with equal probability, and this step is repeated 250 times. By superposing the selected data, a dense array CNT I_D-V_{GS} curve is generated. This entire process is repeated multiple times to obtain a distribution of the projection results. **Figure S11** presents the projected I_{max} and I_{min} at $|V_{DS}|$=0.5 V for different contact lengths.

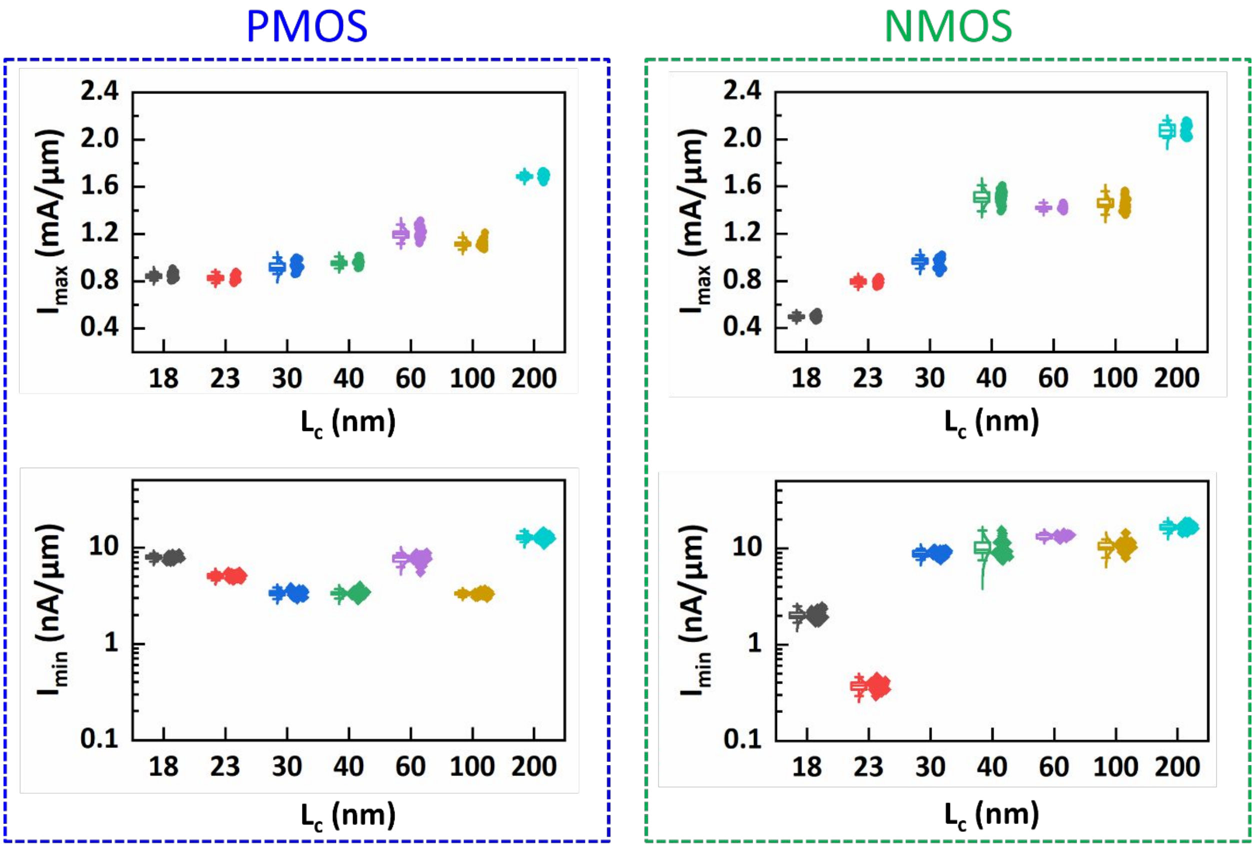

Figure S11. Statistical characteristics of I_{max} and I_{min} at various contact lengths for the projected dense array CNFETs. The CNT density is assumed to be 250 CNT/μm.

(1) Liu, F., Chen, X., Xi, M. *et al.* Comparative study of the extraction selectivity of PFO-BPy and PCz for small to large diameter single-walled carbon nanotubes. *Nano Res.* **15**, 8479–8485 (2022). https://doi.org/10.1007/s12274-022-4425-0.
